# The Novel PKC*θ* from Benchtop to Clinic

**DOI:** 10.1155/2015/348798

**Published:** 2015-05-19

**Authors:** Rouba Hage-Sleiman, Asmaa B. Hamze, Lina Reslan, Hadile Kobeissy, Ghassan Dbaibo

**Affiliations:** ^1^Department of Pediatrics and Adolescent Medicine, Division of Pediatric Infectious Diseases, and Department of Biochemistry and Molecular Genetics, Faculty of Medicine, American University of Beirut, P.O. Box 11-0236, Riad El Solh, Beirut, Lebanon; ^2^Department of Biomedical Science, Faculty of Health Sciences, Global University, P.O. Box 15-5085, Batrakiyye, Beirut, Lebanon; ^3^Department of Biochemistry and Molecular Genetics, Faculty of Medicine, American University of Beirut, P.O. Box 11-0236, Riad El Solh, Beirut, Lebanon

## Abstract

The protein kinases C (PKCs) are a family of serine/threonine kinases involved in regulating multiple essential cellular processes such as survival, proliferation, and differentiation. Of particular interest is the novel, calcium-independent PKC*θ* which plays a central role in immune responses. PKC*θ* shares structural similarities with other PKC family members, mainly consisting of an N-terminal regulatory domain and a C-terminal catalytic domain tethered by a hinge region. This isozyme, however, is unique in that it translocates to the immunological synapse between a T cell and an antigen-presenting cell (APC) upon T cell receptor-peptide MHC recognition. Thereafter, PKC*θ* interacts physically and functionally with downstream effectors to mediate T cell activation and differentiation, subsequently leading to inflammation. PKC*θ*-specific perturbations have been identified in several diseases, most notably autoimmune disorders, and hence the modulation of its activity presents an attractive therapeutic intervention. To that end, many inhibitors of PKCs and PKC*θ* have been developed and tested in preclinical and clinical studies. And although selectivity remains a challenge, results are promising for the future development of effective PKC*θ* inhibitors that would greatly advance the treatment of several T-cell mediated diseases.

## 1. Introduction

Cells respond to environmental stimuli through complex signal transduction pathways. Among key players are the protein kinase C (PKC) family highlighted by numerous studies in regulation of the cell cycle, cancer development, and the stress response [1]. The particular PKC isozyme activated, its cellular localization, and the ensuing protein-protein interactions differentially affect cell survival [2]. Primarily expressed in lymphoid tissues, hematopoietic cells, and muscle cells [3], the novel isozyme PKC*θ* shares its regulatory N-terminal domain and C-terminal catalytic domain with other PKC family members [1]. PKC*θ*, however, plays a unique role in immune responses by modulating multiple molecules such as nuclear factor kappa-light-chain-enhancer of activated B cells (NF-*κ*B), activator protein 1 (AP-1), mitogen-activated protein kinase (MAPK), and c-Jun N-terminal kinases (JNK). Interestingly, it is the only member of the PKC family known to translocate to the immunological synapse between a T cell and an antigen-presenting cell (APC) upon T cell receptor-peptide MHC recognition [4, 5]. PKC*θ* interacts physically and functionally with downstream effectors to mediate T cell activation, differentiation, and migration. In addition to its role in inflammation, PKC*θ* is implicated in certain disorders ranging from autoimmunity, neuroinflammatory diseases, muscular dystrophy, cancer, and diabetes. Here we review experimental studies done on PKC*θ* and their contribution to the development of new therapeutic agents, targeting PKC*θ*, particularly in inflammatory contexts.

## 2. The Structural and Functional Features of PKC Family Members

The PKC family consists of 12 serine/threonine kinases that are divided into three groups based on their corresponding activators/cofactors, conventional (cPKCs), novel (nPKCs), and atypical (aPKCs). The cPKCs include the *α*, *β*, and *γ* isozymes which are activated by Ca^2+^, diacylglycerol (DAG) and tumor-promoting phorbol esters (PMA), in the presence of phosphatidylserine (PS) [6, 7]. The nPKCs (*ε*, *η*, *δ* and *θ*) are activated by DAG and PMA only. The aPKC group includes *ι*, *ζ*, and *μ* kinases which are not activated by Ca^2+^, DAG, or PMA but depend on protein-protein interaction for activation such as p62 in the case of PKC *ζ* [8–10]. An additional group in the PKC family named PKC-related-kinases (PRKs) was also described [11]. This group is also considered the fourth of the PKC family and consists of three members PRK1–3. Like aPKCs, PRKs do not bind Ca^2+^, DAG, or phorbol esters. They are similar in structure to PKCs except for the C1 domain. In addition, PRKs have HR1 motifs which are not present in other PKCs and are presumably responsible for the RhoA binding property of the PRKs.

The structure of protein kinases consists of a regulatory N-terminal domain and a catalytic C-terminal domain held together by a hinge region [12]. Cleavage of the hinge liberates the catalytic domain leading to constitutive activation of PKC. The catalytic domain includes phosphorylation and autophosphorylation sites (discussed later) and, hence, is referred to as the kinase domain. It also contains two highly conserved regions among all PKC isozymes; the C3 element consists of an ATP-binding site and the C4 region is dedicated for substrate binding [12]. On the other hand, the regulatory moiety contains three domains, the pseudosubstrate domain (autoinhibitory sequence), the C1 domain that binds DAG and phorbol esters, and the C2 domain that binds Ca^2+^ [1]. All protein kinases possess the pseudosubstrate domain, but not all isozymes have functional C1 and C2 cofactor binding domains [13]. For instance, cPKCs contain pseudosubstrate, C1 and C2 domains. The nPKCs have pseudosubstrate, C1 and a variant form of C2 domain making it insensitive to Ca^2+^ activation. The atypical PKCs possess a variant form of C1 with the absence of C2 domain [13].

### 2.1. Characteristics of Kinase Catalytic Domain and Pseudosubstrate Domain

The structure of the kinase domain was brought to light when the crystal structure of protein kinase A was first resolved by Knighton and colleagues in 1991 [14]. The ATP and protein substrate bind in the kinase cleft situated between two lobes, consisting of *β*-sheets at the N-terminus and *α* helix on the C-terminus [15, 16]. Before it becomes catalytically competent, yet still inactive, nascent PKCs undergo conformational changes. Such changes occur at three phosphorylation sites that are conserved, among PKC isozymes as well as protein kinases A and G [9]. These sites are located at the activation loop (also referred to as T-loop) positioned at the tip of the kinase domain, at the turn-motif named after the “apex of a turn” structure of the PKA, and at the hydrophobic motif in the C-terminal end of the kinase domain. The order by importance of the phosphorylation starts with the foremost and the rate-limiting phosphorylation at the activation loop by phosphoinositide-dependent kinase (PDK-1) [13, 17–19]. PDK-1 requires phosphatidylinositol-3,4,5-triphosphate for PKC *ζ* phosphorylation [20–22]. In absence of PDK-1, PKC isozymes become prone to rapid degradation before turning into catalytically competent enzymes [23]. The second step of phosphorylation continues with the phosphorylation of the turn-motif (T638 in PKC*α* and S643 in PKC*δ*) followed by phosphorylation of the hydrophobic motif (S657 in PKC*α* and S662 in PKC*δ*) [24–26]. In cPKCs, the turn motif and the hydrophobic motif are autophosphorylated, whereas in nPKCs autophosphorylation occurs only on the turn motif; phosphorylation on the hydrophobic motif is carried out by other kinases. Unlike other PKCs, phosphorylation of the activation loop in PKC*θ* is sufficient for NF-*κ*B stimulation [27]. Studies have shown that mutation at the hydrophobic motif replacing the phosphorylated residue serine by alanine contributes to PKCs thermo-instability [28, 29]. Therefore, the hydrophobic motif, but not the activation loop, is considered a direct mediator for PKC stability [23]. It appears that the hydrophobic motif actually functions as a docking-site for PDK-1 enzyme through its repetitive negatively charged aspartate sequence called PDK-1 interacting fragment (PIF) [16, 30]. This interaction allows PDK-1 to access the activation loop. The atypical PKCs possess an acidic phosphomimetic aspartic acid or glutamic acid in the hydrophobic motif that enhances binding of PDK-1 and phosphorylation of the activation loop [17, 18]. In addition to PDK-1, rapamycin (mTOR) complex 2 (mTORC2) regulates the phosphorylation of the turn motif rather than the hydrophobic motif in cPKC isozymes and novel PKC*ε* [31]. How such phosphorylation protects PKCs from degradation is still not fully understood. However, it is well established that the acidic residues surrounding the activation loop and the binding of the pseudosubstrate post-phosphorylation are essential for enzyme stability [32].

The pseudosubstrate domain is located at the extremity of the regulatory site. It was first described by Kemp and colleagues as a stretch of amino acids that resembles the substrate, except that it contains an alanine residue instead of serine/threonine [33]. A pseudosubstrate has a dual function; it controls both maturation and activation prior to cofactor binding [34]. As mentioned earlier, nascent PKCs need to be phosphorylated to become mature or catalytically competent. Binding of the pseudosubstrate shields the catalytic loop from PDK-1 and prevents its phosphorylation as shown in* in vitro *experiments [35]. Therefore, for PDK-1 to phosphorylate the kinase domain, PKC kinase domain should be in an “open” position devoid of any pseudosubstrate [35]. Once PDK-1 phosphorylates the activation loop, the kinase domain PKC becomes catalytically competent; it undergoes a conformational change indulging the pseudosubstrate to bind at the substrate-binding site. At that point, PKC is said to be “mature” and resistant to phosphatases [35]. For PKC to become catalytically active, upon cofactor binding (DAG, Ca^2+^ and PS), another conformational change displaces the pseudosubstrate from the substrate-binding site giving access to the substrate and allowing subsequent phosphorylation [35].

### 2.2. The Topological Properties of PKCs

The localization of PKC family members in the cell dictates their respective functions. Compartmentalization of PKCs to the membrane is mediated by scaffold/adaptor proteins [34]. Scaffold proteins interact with nascent/competent, mature and active PKC isozymes regulating the kinases' activities either positively or negatively. Examples of scaffold proteins are: receptor for activated C kinase (RACK), substrates that interacts with C kinase (STICK), receptor for inactive C kinase (RICK), and A-kinase activating protein (AKAP) [34]. RACKs and STICKs bind to active PKCs whereas AKAPs and RICKs interact with inactive PKCs. Binding of RACK increases the phosphorylation capacity of PKCs several-folds independently from the substrate identity [36]. However, STICK itself acts as a substrate for PKC in addition to its function as an anchoring protein [36, 37]. Caveolins represent another group of scaffold that helps PKC*α* and PKC*ζ* translocate to the caveolar microdomains where they are subsequently activated [38]. AKAP79 recruits PKC isozymes to the postsynaptic dendritic fraction rendering them inactive [39]. Several other scaffold proteins such as CARMA1 (CARD-containing MAGUK protein 1), 14-3-3*τ*, and Vav1 are particularly involved in regulating PKC*θ*'s translocation and activation and will be discussed later in the manuscript.

### 2.3. Termination of the Kinase Activity in PKCs

The kinase activity of PKCs is terminated by dephosphorylation [40]. However, this process takes place when protein kinases are in an “open” conformation, in other words, when the kinase domain is unbound by the pseudosubstrate or when a particular PKC is constitutively active [41]. For cPKCs and nPKCs, dephosphorylation is carried out by the PP2C member PHLPP (pleckstrin homology domain leucine-rich repeat protein phosphatase) at the hydrophobic motif and by PP1/PP2A protein phosphatases at the turn motif [40, 42–44]. In other contexts, the effect of phosphatases on PKCs is indirect. For instance, the dephosphorylation of PKC*θ* downstream molecules, CARMA1, by PP2A leads to PKC*θ* deactivation [45]. Hence, dephosphorylation predisposes “naked” protein kinases to ubiquitination and degradation [46, 47]. There are two types of ubiquitination, proteasomal and lysosomal ubiquitination. The former requires multiple ubiquitin tags while the lysosomal pathway involves a monoubiquitination [48]. Many PKC isozymes, including *α*, *δ*, and *ε*, undergo proteasomal ubiquitination in response to tumor-promoting phorbol ester 12-*O-*tetradecanoylphorbol-13-acetate (TPA). Other protein kinases undergo lysosomal ubiquitination such as PKC*θ* (discussed in the next section) and PKC*ε*. Importantly, ubiquitination not only mediates protein degradation but can also modify the kinase activity. Indeed, monoubiquitinated PKC*ε* promotes IKK*β* phosphorylation, thereby triggering tumorigenesis [49].

## 3. A Novel PKC Isoenzyme, PKC***θ***

### 3.1. Structural Domains of PKC*θ*

**Figure 1:**
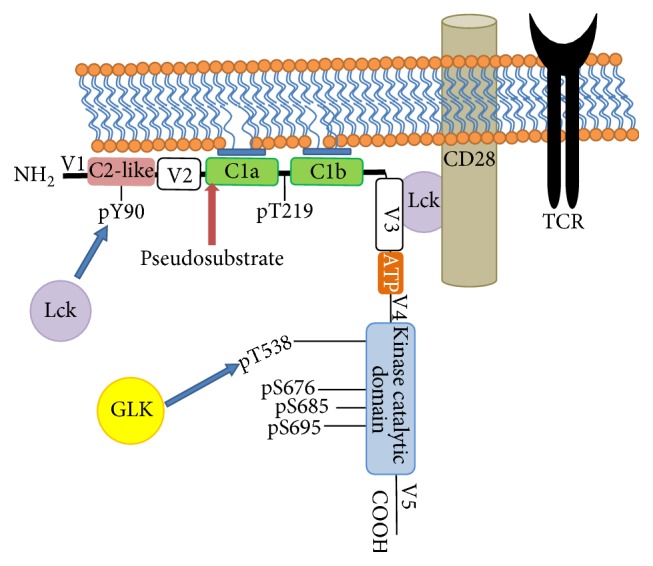
A schematic model of membrane-associated PKC*θ* in TCR/CD28 stimulated T cells. Regulatory N-terminal domain consists of V1, C2-like, V2, C1a, C1b, and V3 domains. Catalytic C-terminal domain consists of ATP binding site, V4, kinase catalytic domain (substrate binding site), and V5. PKC*θ* binds to the membrane through diacylglycerol by its C1a and C1b domains. It interacts with CD28 via Lck through its V3 domain. Blue arrows represent phosphorylation by respective enzymes on specific amino acid residues. V: variable domain; C: constant domain; GLK: germinal center kinase- (GSK-) like kinase; Lck: Lymphoid cell kinase; TCR: T cell receptor.

Primarily expressed in lymphoid tissues and hematopoietic cells [50], PKC*θ* is a single polypeptide kinase composed of 706 amino acids that typically phosphorylates serine or threonine residues. PKC*θ* shares its structure with other PKC family members; it contains a regulatory N-terminal domain and a C-terminal catalytic domain tethered together by a hinge region as seen in Figure 1 [1]. The regulatory domain of PKC*θ* consists of the C2-like domain sequence, similar to the Ca^2+^-binding C2 domain of other PKCs, except that it does not really bind Ca^2+^. The C2-like domain allows PKC*θ* to interact with a receptor for activated C kinase (RACK) which regulates its translocation to the membrane [49]. In addition to the C2-like domain, the regulatory domain of PKC*θ* includes C1a and C1b domains that have diacylglycerol (DAG) and phorbol esters binding sites [51]. The C1b domain has much higher affinity for diacylglycerol than the C1a domain [52]. The pseudosubstrate region in the C1a domain consists of a small sequence of amino acids that mimics a substrate and binds to the substrate-binding cavity in the catalytic domain [33]. However, this sequence lacks phosphorylatable serine and threonine so it prevents access of substrates to the catalytic domain and keeps the enzyme inactive. Moreover, regulatory domains include the variable V1, V2 and V3 domains. The V3 domain, with its proline-rich motif, is unique to PKC*θ*, essential and sufficient for its translocation to immunological synapses by linking it to CD28 receptor via the kinase Lck [53, 54].

The crystal structure of PKC*θ* catalytic domain has been published in 2004 [55] revealing an N-terminal lobe and a C-terminal lobe. The catalytic C-terminal domain consists of an ATP binding site, V4, substrate binding site, and V5. ATP binds to a glycine-rich loop (GXGXXG) at the interface of the two lobes while the substrate binds to an *α*C helix. Additionally, important elements of the conserved catalytic domain include a kinase activation loop with phosphorylatable threonine 538 (pT538), a hydrophobic motif containing phosphorylatable serine 695 (pS695), and a turn motif containing conserved phosphorylatable serine 676 (pS676) and phosphorylatable serine 685 (pS685) [55]. The catalytic domains of PKCs are highly conserved, with the exception of the variable V5 region consisting of 60–70 amino acids. This variable domain highly contributes to the regulation of PKC*α* activity through multiple mechanisms; by stabilizing the kinase through direct interactions with its N-lobe, by interacting with the pseudosubstrate in the N-terminal regulatory domain and by mediating subcellular localization through interaction with RACK [56]. Nothing has been published yet on the role of the V5 domain in PKC*θ* isozyme.

### 3.2. Physical and Functional Interactions of PKC*θ* with Substrates and Regulators

PKC*θ* can interact either physically or functionally, activating or synergizing with the activity of other proteins. Many examples will be summarized in this section starting with T cells proteins. The 14-3-3 family proteins were described as potential regulators of PKCs [57]. These proteins associate with several protooncogene and oncogene products modulating their activity. 14-3-3*τ* isoform is highly expressed in T cells and associates with PKC*θ in vitro* and in intact T cells. 14-3-3*τ* binds directly to PKC*θ* in the cytosol, preventing its activation and translocation to the membrane [57]. When overexpressed, it can also inhibit the enzymatic activity of PKC*θ* by blocking its association with substrate and/or ATP. A direct interaction between PKC*θ* and SAP (SLAM-Associated Protein) was also described in T cell activation signaling [58, 59]. SAP mediates the recruitment and activation of the protein kinase Fyn that, in turn, phosphorylates SLAM (Signaling Lymphocyte Activation Molecule). Phosphorylation of SLAM creates docking sites for many proteins and enzymes such as PKC*θ*, leading to NF-*κ*B activation [58, 59]. It was also shown that SAP constitutively associates with PKC*θ* in T cells via arginine 78 of SAP, independently of Fyn, but via the formation of a ternary SLAM/SAP/PKC*θ* complex following T cell activation [60].

Interestingly, an E3 ubiquitin ligase, Casitas B-lineage lymphoma (Cbl-b) was described to suppress T cell activation when mediated by TCR signaling alone without CD28 costimulatory signals [61]. Upon costimulation with CD28, however, the suppression of T cell activation is removed since Cbl-b gets degraded in a mechanism that depends on the activity of PKC*θ* [62]. Furthermore in T cell context, the protooncogene Vav, a GDP/GTP exchange factor (GEF), was also described to associate with PKC*θ* in thymocytes in response to TCR-mediated apoptosis [63]. PKC*θ* was found to synergize with Vav for the activation of NF-*κ*B [64]. It is likely that Vav helps in the translocation of PKC*θ* to synaptonemal microdomains leading to their colocalization and T cell activation [65]. It remains to be proved whether Vav translocates to the membrane following PKC*θ* phosphorylation or by direct contact with PKC*θ* [66], especially since the interaction between Vav and PKC*θ* appears to be a functional rather than a physical association [65]. In addition to SAP and Vav, CARMA1 is inducibly phosphorylated on S552 of its linker region by PKC*θ* upon TCR-CD28 costimulation. This phosphorylation mediates TCR-induced NF-*κ*B activation [67]. Furthermore, it was shown that CARMA1 acts to contribute to the upregulation of the protein mucin in response to the bacterium* Haemophilus influenzae* and phorbol ester PMA in respiratory epithelial cells via a PKC*θ*-MEK-ERK pathway [68]. Other interaction mechanisms remain unclear such as the potential interaction between PKC*θ* and interleukin-2-inducible T-cell kinase (Itk) in T lymphocyte signal transmission [69].

In addition to its roles in regulating the activation and proliferation of lymphocytes, PKC*θ* appears to have an important role during muscle histogenesis [70]. Recent studies showed that PKC*θ* is essential for cardiomyocytes survival and cardiac tissue remodeling by preventing cardiomyocytes' death upon extensive work [71]. In skeletal muscle models, it was not understood why embryonic myoblasts differentiate in the presence of transforming growth factor beta (TGF beta) while fetal myoblasts do not. It was found that PKC*θ* is selectively expressed in fetal skeletal myoblasts but not in embryonic skeletal myoblasts [70]. Embryonic myoblasts lacking PKC*θ* did not respond to TGF beta or differentiate in its presence. However, the sensitivity of fetal myoblasts to the inhibition of differentiation exerted by TGF beta is mediated by the expression of PKC*θ* in these cells [70]. Recently, PKC*θ* was found to regulate profusion genes caveolin-3 and *β*1D integrin and induce focal adhesion kinase phosphorylation resulting in mononucleated myoblasts fusion and formation of multinucleated myofibers [72]. In this context, RACK1 acts as an adapter between PKC*θ* and integrins [73]. Another study shed light on the involvement of PKC*θ* in endothelial cell migration via integrins [74]. It described a novel 20 kD protein, theta-associated protein or TAP20 whose transcription depends enzymatically on active PKC*θ* [74]. TAP20 directly interacts with the cytoplasmic tail of the *β*5 integrin subunit, thus interfering with the integrin-cytoskeleton interaction required for focal adhesion formation [74]. Furthermore, PKC*θ* was shown to mediate the binding of leukocyte function-associated antigen 1 (LFA-1) on T cells to immunoglobulin-like cell adhesion molecule 1 (ICAM-1) on APCs following T cell activation [75]. In this context, PKC*θ* associates with RapGEF2 which facilitates Rap1 activation and subsequent surface distribution of LFA-1 [76]. The relocation of LFA-1 and its conformational change increase its binding affinity to ICAM-1 [77]. Moreover, the clusters of LFA-1 on the surface induce actin polymerization and remodeling, thereby enhancing T cell adhesion [78]. Cytoskeletal remodeling also involves the microtubule cytoskeleton where the microtubule-organizing center (MTOC) becomes oriented towards the APC to enable efficient cargo trafficking toward the APC [79]. Interestingly, it was shown that PKC*θ* was required for MTOC reorientation [80]. In another context, PKC*θ* was found to be involved in spectrin-based cytoskeleton remodeling during apoptosis. Spectrin, which is known to link the cell membrane to the actin cytoskeleton, aggregates with PKC*θ* in the early stages of apoptosis [81]. Notably, a unique role of PKC*θ* was revealed in intestinal epithelial monolayers where active PKC*θ* directly phosphorylates tubulin monomers promoting their assembly into microtubules and increasing microtubule stability [82]. Hence, it was shown that loss of PKC*θ* affects the cytoskeletal integrity leading to an increase in epithelial barrier permeability, a symptom of intestinal inflammation.

### 3.3. PKC*θ* in the Immunological Synapses and Lipid Rafts

PKC*θ* is highly expressed in leukemic Jurkat T cells [83]. It is the only member of PKC family to be recruited to the immunological synapse in effector T cells [4]. Immunological synapses form between a T cell and an antigen-presenting cell (APC) following T cell receptor-peptide MHC recognition [4, 5]. It is composed of a central supramolecular activation cluster (cSMAC) surrounded by a peripheral supramolecular activation cluster (pSMAC). It was found that accumulation of lipid rafts in immunological synapses does not increase upon TCR/CD28 stimulation; they rather reorganize preferentially in the cSMAC instead of pSMAC [84]. PKC*θ* appears to be recruited to the junction between the cSMAC and pSMAC in a CD28 costimulatory-dependent manner [85, 86], more specifically by physical association with the cytoplasmic tail of CD28 [54]. Many studies investigated the mechanism by which PKC*θ* translocates to the immunological synapses and revealed that it partially depends on phospholipase C activity and DAG production but also on a novel signaling pathway [85, 87]. It was proposed that such translocation is mediated by the PKC*θ* regulatory V3 domain and requires Lck [88]. In addition to Lck, all of Vav1, phosphatidylinositol 3-kinase (PI3-K), the small GTPase Rac, and actin cytoskeleton reorganization participate in regulating the membrane localization and consequent activation of PKC*θ* [87, 89]. In addition to the regulatory domain, the kinase domain is of great importance with respect to the immunological synapse localization of PKC*θ*. An active kinase domain permits the retention of PKC*θ* in the immunological synapse, likely via autophosphorylated sites that are still undefined [90].

### 3.4. Role of PKC*θ* in Interleukin-2 Production during T Cell Activation

Upon TCR and CD28 costimulation, fully activated PKC*θ* plays an important role in mediating signaling events that lead to the activation of transcription factors such NF-*κ*B, AP-1 and NF-AT. The NF-*κ*B signaling pathway is the major target of PKC*θ* in T cell activation that leads to interleukin-2 (IL-2) production. NF-*κ*B is usually present in the cytosol in an inactive form whereby its nuclear localization sequence is shielded by inhibitors such as I*κ*Bs [91, 92]. These inhibitors, when phosphorylated by PKC*θ*-activated IKKs, undergo degradation resulting in NF-*κ*B translocation to the nucleus where it regulates gene transcription of IL-2. The activation of IKKs by PKC*θ* mediated by multiple effectors such as CARMA1 [67], discussed above. Another study revealed a direct interaction between PKC*θ* and IKK*β* that shed light on a different potential pathway linking PKC*θ* to NF-*κ*B [93]. AP-1, a dimer of Jun and/or Fos proteins is also a transcription factor that regulates IL-2 production. PKC*θ* activates SEK1, a MAP kinase that phosphorylates and activates JNK, which then activates Jun [94]. A third pathway involving NF-AT is also thought to be essential for full T cell activation, although cross-talk exists between the different PKC*θ*-dependent IL-2 production pathways [95]. Activation of T cells promotes activation of phospholipase C, which triggers the formation of the two second messengers, inositol triphosphate (IP3) and DAG. IP3 causes the elevation of cytosolic Ca^2+^, which activates the Ca^2+^-dependent serine/threonine phosphatase, calcineurin [95]. It was initially thought that PKC*θ* regulates IL-2 through TCR downstream effectors; however, later studies revealed that in PKC*θ*-deficient mice, IP3 production was reduced thereby leading to defective Ca^2+^ response and NF-AT transactivation [95, 96]. Such defect in Ca^2+^ mobilization is likely due to the lack of enzymatic activation and subsequent membrane association of PLC [95]. These findings suggest an unforeseen role of PKC*θ* as an upstream regulator of phospholipase C (PLCgamma1) via tyrosine kinase Tec [96].

### 3.5. Regulation of PKC*θ* Translocation to Lipid Rafts and Activation

PKC*θ* acts as a kinase receptor for phorbol esters and DAG to mediate many cellular responses. Hence, PKC*θ* is regulated by certain lipids, phosphorylation, and ubiquitination. First, lipids modulate PKC*θ* activity by cofactors such as DAG. The binding of DAG enhances the interaction between PKC*θ* and the acidic phosphatidylserine [97] which decreases the binding affinity of the pseudosubstrate inhibitor and leads to PKC*θ* activation as discussed earlier. Second, PKC*θ* activity is regulated by phosphorylation and autophosphorylation mechanisms in which many kinases participate to allow the translocation of PKC*θ* to the membrane. Lck directly phosphorylates PKC*θ* at Y90, which stimulates NF-AT and NF-*κ*B activation in T cells [88, 98]. Lck binding regulates membrane translocation of PKC*θ* by forming of PKC*θ*/Lck/CD28 complex [53, 99, 100]. It is still unknown whether or not the Y90 phosphorylation has a direct influence on both the formation of the above complex and PKC*θ* catalytic activity. Furthermore, it appears that germinal center kinase-like kinase (GLK) phosphorylates PKC*θ* on T219, a novel S/T residue, and thereafter regulates its translocation to the lipid rafts upon TCR stimulation [101]. Indeed, T219 phosphorylation induces localization of PKC*θ* to lipid rafts and the immunological synapse, allowing it to activate downstream effectors in TCR signaling, independent from its kinase activity [102].

As for the role of autophosphorylation, T538, S676, S685 and S695 are important regulation sites at the catalytic domain of PKC*θ* [27, 103]. The PKC*θ* autophosphorylation sites are interdependent in that when T538 phosphorylation site is lost, the remaining sites S676 an S695 become more susceptible to dephosphorylation by phosphatases [27]. T538 is a critical site that regulates PKC*θ* kinase activity and T cell activation [27] but does not seem to influence PKC*θ* translocation to lipid rafts [102]. Constitutive autophosphorylation of T538 occurs at the activation loop where substrates and cofactors bind near the active site of the kinase domain [104]; this step helps retain the active conformation of PKC*θ* [55]. Additionally, GLK directly associates with PKC*θ* in T cells upon anti-CD3 stimulation and phosphorylates the T538 residue [101]. Such phosphorylation at the turn motif contributes to the regulation of the enzyme's catalytic activity by stabilizing its active conformation [105, 106]. PKC*θ*'s S676 site is constitutively autophosphorylated and its phosphorylation is moderately increased upon anti-CD3/CD28 costimulation [107]. How this phosphorylation affects the activity of PKC*θ* and downstream NF-*κ*B activation remains controversial. Conversely, autophosphorylation of PKC*θ* on S685 appears to regulate the function of PKC*θ* and T cell activation during TCR signaling [103]. S695 is a constitutive autophosphorylation site in the C-terminal hydrophobic motif of PKC*θ* is likely induced by CD3 stimulation [89, 107, 108]. Interestingly, PKC*θ* S695A mutant results in great loss of T538 phosphorylation status [98, 103]. Hence, S695 phosphorylation is required for optimal PKC*θ* activation and T cell activation during TCR signaling [27, 102, 103] but its role in the regulation of translocation of PKC*θ* to the membrane is still controversial [89, 108].

As mentioned earlier, PKCs are regulated by degradation following ubiquitination. Upon sustained Ca^2+^ and calcineurin signaling, a state of anergy or antigen unresponsiveness is induced in T cells mediated by proteolytic degradation of PKCs [109]. Indeed, it was shown that PKC*θ* goes through lysosomal ubiquitination by activation of myriad proteins. Among these proteins is Itch, the endosome-associated E3 ligase, which catalyzes the ubiquitination and ligation of monoubiquitinated PKC*θ* to Tsg101 receptor, a component of ESCRT-1 complex located on lysosomal vesicles [110].

## 4. PKC***θ*** Mechanisms of Action in Various Pathologies

Perturbations of PKC*θ* activity can result in a variety of diseases and disorders including immunological disorders such as autoimmune and inflammatory diseases, cancer, and diabetes. In the following section, we will summarize PKC*θ* mechanisms of action in various pathologies.

### 4.1. Autoimmune Responses and Inflammation

PK*θ* is highly expressed in some immunological disorders and conditions with inflammation. Indeed, PKC*θ* plays a dual role in inflammation through its differential regulation of effector T cells (T^effs^) and regulatory T cells (T^regs^) [5, 111]. The renowned translocation and function of PKC*θ* at the immunological synapse actually occurs in T^effs^, either CD4^+^ or CD8^+^ T cells, as it promotes their proliferation to mediate inflammation [5]. In T^regs^, however, PKC*θ* is sequestered away from the immunological synapse and this allows T^regs^ to suppress the activity of T^effs^ in order to maintain balance of immune reactions, provide tolerance to self-antigens, and prevent autoimmunity [111–113]. Hence, increased PKC*θ* activity has become a hallmark of autoimmune disorders, which result from activation of self-reactive T cells that differentiate into effectors and attack self-tissues [114]. Additionally, overexpression of the PKC*θ*-activator GLK enhances PKC*θ* activity and subsequent stimulation of IKK leading to autoimmunity in systemic lupus erythematosus [101]. This is also true in patients with rheumatoid arthritis where GLK expression was significantly higher in their peripheral blood T cells compared to healthy subjects, and it colocalized with phosphorylated PKC*θ* in T cells [115].

Therapeutically, the inhibition or suppression of PKC*θ* helps protect cells from autoimmune disorders. For instance, PKC*θ*-deficient mice show diminished severity, articular cartilage damage, and bone destruction from Th1-dependent antigen-induced arthritis compared to wild-type mice [116]. This could be due to the reduced expression of the cytokines IFN-*γ*, IL-2, and IL-4 in their CD4^+^ T cells [116]. Moreover, PKC*θ*−/− mice immunized with myelin oligodendrocyte glycoprotein are also resistant to development of autoimmune encephalomyelitis, a model for multiple sclerosis. CD4^+^ T cells from these mice became primed and accumulated in secondary lymphoid organs in the absence of PKC*θ*, with severely diminished IFN-*γ*, TNF, and IL-17 production [117–119]. PKC*θ* is also required for autoimmune hepatitis induced by concanavalin A, which normally activates CD1d-positive NK cells, rapidly resulting in the generation of the cytokines IFN-*γ*, IL-6, and TNF-*α* in large amounts that induce liver damage [120, 121]. In another model, immunization of PKC*θ*-deficient mice with myosin peptide revealed that these animals fail to develop autoimmune myocarditis as well as the IL-17-producing CD4^+^ cells (Th17) which mediate the disease [122]. In fact, PKC*θ* promotes differentiation of T helper 17 (Th17) cells through up-regulation of transcription factor Stat3 through NF-*κ*B and AP-1 upon TCR signaling [123].

Moreover, PKC*θ* is crucial for* in vivo* development and harmful immune responses of Th2 cells including pulmonary hyperresponsiveness and allergic reactions to inhaled allergen in a model of asthma [124, 125]. However, PKC*θ* is somewhat dispensable for Th1-mediated responses as it only affects Th1 initial development, but its deficiency does not impair their activation or cytokine production, especially under conditions that involve strong Th1-inducing stimuli [125]. In allogeneic bone marrow transplantation, PKC*θ* promotes graft-versus-host-disease (GVHD), which is a potentially lethal complication caused by alloreactive donor T cells that recognize mismatched major histocompatibility molecules [126]. However, in the absence of PKC*θ*, T cell responses triggered in mice by viral infection or administration of an antigen were relatively normal, and the graft-versus-leukemia effect was preserved [126]. PKC*θ* is also necessary for survival of alloreactive T cells responsible for allograft rejection through up-regulation of the anti-apoptotic protein, Bcl-xL [114, 127]. Taken together, this evidence suggests that inhibition of PKC*θ* under such conditions may result in more successful transplants due to long-term tolerance of grafts [121, 128].

In addition to its role in regulating autoimmune and immunosuppressive responses, PKC*θ* is involved in many inflammatory diseases such as nervous and muscular inflammatory diseases. First, PKC*θ* is involved in inflammatory brain conditions that result in blood-brain barrier dysfunction [129, 130]. The central molecule in such diseases is the proinflammatory interleukin-1beta (IL-1*β*) which induces activation of PKC*θ* and subsequent phosphorylation of the tight junction protein* zona occludens* (ZO)-1 thereby reducing transendothelial electrical resistance as is seen in barrier leakage [130]. Second, inflammation is also a major detrimental factor in muscle dystrophy that promotes muscle degeneration thereby obstructing healing. In this context, PKC*θ* is the suspected player though its pro-inflammatory role [131, 132]. Knockdown of PKC*θ* in a mouse model of Duchenne muscular dystrophy indeed prevented muscle wasting and enhanced regeneration and performance of muscle tissue [132].

### 4.2. Cancer

As previously mentioned, PKC*θ* is essential for T cell proliferation as it induces expression of IL-2 through NF-*κ*B and AP-1. In addition, PKC*θ* mediates one of the mechanisms by which leukemic T cells are protected from Fas-induced apoptosis by phosphorylating the bcl-2 family protein BAD [83, 133]. PKC*θ* is also involved in tumor development. For example, it is a downstream player in pre-TCR-Notch3 signaling where its activation of NF-*κ*B is responsible for the development of Notch3-dependent T-cell lymphoma [134].

Moreover, upon pre-TCR activation, PKC*θ* prevents Notch3 degradation by regulating the phosphorylation and localization of E3 ubiquitin ligase c-Cbl [135]. PKC*θ* is positively associated with breast cancer cell proliferation and invasion [136, 137]. PKC*θ* activates Akt, which in turn reduces activity of forkhead box O protein 3a (FOXO3a) and expression of its target genes estrogen receptor *α* (ER*α*) and p27 [136]. This pathway results in depression of the transcription factors NF-*κ*B and c-Rel, which are highly implicated in mammary tumorigenesis [136]. In such ER-negative cells, enhanced PKC*θ* signaling also leads to the activation of ERK1/2 and Ste20-related proline-alanine-rich kinase (SPAK) as well as the phosphorylation of the Fos family protein Fra-1, thereby stabilizing it and regulating its role in the progression and maintenance of invasive breast cancer cell lines [137]. In addition to leukemia and breast cancer, gastrointestinal stromal tumors (GISTs), the most common mesenchymal tumors, are characterized by high expression and activation of PKC*θ* [138–140]. PKC*θ* is used as a marker for diagnosis of KIT protein-negative GIST [138, 141]. Knockdown of PKC*θ* inhibits cyclin A expression but causes the overexpression of the tumor suppressors p21, p27, and p53 resulting in cell-cycle arrest and apoptosis of GIST48 cells [140].

PKC*θ* plays a central function in the resistance to tumor development through its role in promoting T cell survival [142, 143]. It was found that up-regulation of sarco/endoplasmic reticulum Ca^2+^-ATPase 3 (SERCA3) by tumor environment inhibits PKC*θ* in human CD4^+^ T and causes retention of NF-*κ*B in the cytosol, leading to apoptosis of these T cells [143]. Studies in PKC*θ*-deficient mice demonstrated the importance of PKC*θ* in the immune response to leukemia as these mice had higher incidence and faster onset of the disease than wild-type mice [144]. PKC*θ* is also expressed in natural killer (NK) cells and is considered critical for NK-cell mediated anti-tumor surveillance [145, 146]. Development of MHC-I-deficient tumor* in vivo* is more likely in PKC*θ*−/− mice than in wild-type mice; such phenotype was associated with reduced NK recruitment and activation [145]. In fact, PKC*θ* phosphorylates WASp-interacting protein (WIP), which is central for the formation of the protein complex required for NK cytotoxic activity [147]. NK cell-activating receptors also require PKC*θ* for intracellular signaling that leads to generation of IFN-*γ* [148].

### 4.3. Diabetes and Insulin Resistance

PKC*θ* is the mediator between lipid metabolism and insulin resistance, which is a leading cause of type 2 diabetes mellitus [149, 150]. Elevation in plasma free fatty acids levels increases intracellular fatty acyl-CoA and DAG which in turn activates PKC*θ* in skeletal muscle which phosphorylates S307 on insulin-stimulated insulin receptor substrate 1 (IRS-1) resulting in reduced tyrosine phosphorylation and IRS-1-associated PI3-kinase activity [151–153]. This event leads to insulin resistance by alleviating insulin-stimulated muscle glycogen synthesis. Similar effects of PKC*θ* were observed in adipose tissue and the liver [154–156]. A more recent study has actually proposed PDK-1 as a direct target of PKC*θ* in insulin resistance, in a pathway independent from IRS-1/2 [157]. PKC*θ* negatively regulates insulin receptor activation of PDK-1 by S504/332 phosphorylation, thereby inhibiting PDK-1-mediated Akt phosphorylation and subsequent PI-3K signaling. Up-regulation of PKC*θ* that is inversely proportional to insulin sensitivity has also been reported in type 2 diabetic subjects [158]. Furthermore, PKC*θ* expression in critical regions of the amygdala and hypothalamus is linked to diet-induced obesity and reduced insulin signaling at the level of the central nervous system response [159–162].

## 5. PKC***θ*** as Target in Clinic

Activation of T cells presents the initiating event in immunological disorders and plays an important role in regulating the immune response. Isozyme-specific perturbations in PKC activity have been identified in numerous human diseases [163]. Therefore, the modulation of PKC activity presents an attractive approach for clinical drug development. Accordingly, agents that inhibit PKCs could contribute to the suppression of immune responses to achieve successful transplants and to prevent many immunological disorders resulting from autoimmune and inflammatory diseases. Many hurdles challenge the development of kinase-specific inhibitors including potency, and selectivity. Most of the PKC domains show high sequence and structural similarity among the isoforms, making it difficult to design molecules that selectively target each isoform. Furthermore, the high degree of homology in the kinase region among the more than 500 kinases in the human genome makes the design of a PKC inhibitor targeting the kinase domain of interest a major challenge [164, 165]. Moreover, PKCs isoforms have revealed many complex interrelationships and interactions. For example, one particular isoform may be involved in different diseases. Several isoforms may be involved in one particular disease, while for a particular disease two PKC isoforms may produce contrary effects. For instance, PKC*α* and PKC*δ* play opposite roles in the proliferation and apoptosis of glioma cells [166].

### 5.1. PKC Inhibitors and the Clinical Trials

Inhibitors of PKC can be classified according to their sites of interaction within the PKC protein structure [163]. Inhibitors of the catalytic domain are directed to either the substrate site or ATP-binding site whereas inhibitors of the regulatory domain may target the phospholipid or phorbol ester binding site by mimicking diacylglycerol [167]. Moreover, inhibitors that disrupt protein-protein interactions at a specific subcellular location or with a specific substrate may provide a new approach to selectively inhibit the phosphorylation of substrates between unique regions in each PKC and its corresponding interacting protein or substrate [163]. Although a wealth of inhibitory compounds is available, few demonstrate specificity for either PKC alone or individual PKC isoforms. Many research efforts are underway to develop PKC-based drugs with several compounds currently in clinical trials.

The best characterized ATP-competitive small molecules are the bisindolylmaleimides [168]. These water-soluble compounds bind to the ATP-binding pocket and limit phosphorylation. The classic example, staurosporine, has pan-PKC activity, binding to all isozymes as well as several other serine/threonine kinases [169]. The experimental and docking interactions of staurosporine with PKC*θ* displayed important hydrogen bonding with different amino acid residues of the PKC*θ* active site [163]. In fact, staurosporine is one of the most powerful PKC inhibitors in* in vitro* models [163]. However, its poor kinase selectivity hampered its further development, prompting efforts to synthesize more PKC-selective analogues. Among these are 7-hydroxystaurosporine or UCN-01 [170] and N-benzoyl-staurosporine [171], which have less PKC-inhibitory activity than the parent compound, but a higher degree of PKC selectivity when assayed for inhibition of different kinases [172]. However, these agents display specificity against conventional isoforms of PKC over novel Ca^2+^ independent isoforms. Sotrastaurin (AEB071) is a PKC inhibitor that has strong and specific activity against PKC*θ*, PKC*α*, and PKC*β* and lesser effect on PKC*δ*, PKC*ε*, and PKC*η*, suggesting that sotrastaurin would inhibit not only T cells, but also a variety of other cells. It inhibits more than 200 other kinases, including those important for early T cell activation, such as Lck. Sotrastaurin acts through PKC to inhibit T-cell activation that is initiated by the binding of peptide-MHC complexes and CD28 costimulation [173, 174].* In vivo* data from rodents and nonhuman primates confirmed the potential of sotrastaurin in preventing allograft rejection and reducing the inflammatory response [175, 176]. Results from an initial clinical trial in patients with psoriasis showed improvements in clinical and histological assessments [177]; however, data from early trials in kidney transplant recipients were less encouraging. Sotrastaurin is currently used as an immunosuppressant in phase I trials for liver transplantation [178], and phase II trials for renal transplantation [179]. Although sotrastaurin appears to be well-tolerated based on published clinical trial data, long-term data is needed to confirm the safety and efficacy profile of this novel compound. Efforts to develop a more selective inhibitor led to the discovery of enzastaurin [180–183] and ruboxistaurin [184], which are more selective for PKC*β* over other isozymes. Furthermore, Midostaurin (also known as PKC412 or n-benzoylstaurosporine) exhibits improved selectivity for PKC-ATP binding sites, but shows modest isozyme specificity [185, 186]. These inhibitors are undergoing clinical trials. As for enzastaurin, phase I studies showed prolonged disease stabilization in patients with lung cancer, colorectal carcinoma and renal carcinoma [187]. Ongoing clinical trials of enzastaurin alone or in combination with conventional chemotherapies are being investigated in recurrent brain tumor (Phase I), advanced or metastatic malignancies (Phase II), prostate cancer (Phase II), breast cancer, ovarian cancer, and peritoneal cavity cancer [188]. Concerning Ruboxistaurin, it has shown efficacy in the treatment of diabetic retinal and renal abnormalities both in preclinical and human studies [189]. Midostaurin was well-tolerated in phase I study in patients with malignant melanoma but unfortunately phase II trial failed to demonstrate significant clinical activity [185].

The best characterized compound targeting the activator binding C1 domain is bryostatin-1 [190]. Bryostatin-1 is a partial agonist of several members of the PKC family [191]. The binding of bryostatin-1 to PKC results in PKC activation, autophosphorylation, and translocation to the cell membrane [190]. Bryostatin-1-bound PKC is then downregulated by ubiquitination and degradation in proteasomes [190]. Bryostatin-1 is expected to modulate classical PKC isoforms associated with Ca^2+^ signaling as well as novel isoforms independent of Ca^2+^ [190]. Bryostatin-1 has been investigated for anticancer activity in phase I and II clinical trials using a wide range of tumor types [192, 193] and showed promising activity in the treatment of refractory acute leukemia and indolent hematologic malignancies [194–196]. However, several phase II studies were disappointing in melanoma [197], colorectal cancer [198], and gastric carcinoma [199]. Moreover, bryostatin-1 has demonstrated significant chemosensitizing activity when combined with conventional therapeutics including arabinofuranosylcytosine [200], tamoxifen [201], fludarabine [202], taxol [203] in leukemia cells. Protection of PKC from being downregulated by the strong ligand, phorbol ester, led to the design of selective PKC-binding bryostatin analogues. These molecules show selectivity in binding to the C1 domain of various PKC isozymes and may represent a novel class of PKC regulators [204].

### 5.2. PKC*θ* Inhibitors in Preclinical Studies

A large number of PKC*θ* inhibitors have been reported. These can be classified on the basis of their parent scaffolds, such as aminopyrimidine, pyridine carbonitrile (phenyl, furan, benzofuran, benzothiophene and vinyl phenyl analogs) and thieno (2,3-b) pyridine-5-carbonitriles (2-alkenyl and 2-phenyl) derivatives (2-phenyl and 4-amino indole modification) (for chemical structures, refer to review [194]). Compounds belonging to the amino pyrimidine class are the first discovered inhibitors of PKC*θ* and are considered more selective than members of any other category [205].

Different derivatives have been developed by making appropriate modifications in groups R1, R2 and R3 [206]. For instance, R1 may be substituted by NO_2_ and CF_3_ groups; R2 may be substituted by cyclohexane ring whereas R3 by some bulkier groups like 2-bromo benzylamine, 2-chloro benzylamine. The group substitution of amino pyrimidine derivatives can affect its inhibitory activity. For example, the replacement of some groups such as nitro (–NO_2_) with CF_3_ group decreases the activity of molecules by ten times; whereas the presence of the nitro (–NO_2_) group at the 5th position and substitution of hydrogen atom of amino group at the 2nd position with 2-bromobenzylamine, 2-SCH_3_ benzylamine and 2-SCF_3_ benzylamine group increases the potency of molecules in comparison with other substitution groups [207]. Moreover, the stereoisomerism and the geometric isomerism (cis, trans) can affect the biological activity of inhibitors. The pyridine carbonitrile category of PKC*θ* inhibitors consists of C-5 substituted 3-carbonitrile pyridine derivatives. In the derivative inhibitors, C-4 and C-5 positions are substituted with amino indole and different kinds of heteroaryl/aryl groups, respectively [207, 208]. On the basis of substituents at C-5 position, different derivatives have been developed like phenyl, furan, benzofuran, benzothiophene and phenyl vinyl analogues of pyridine carbonitrile. A series of 5-phenyl-3-pyridinecarbonitriles [209], 5-vinyl-3-pyridinecarbonitriles [210], 5-vinyl phenyl sulfonamide-3-pyridinecarbonitriles [211], 5-vinylaryl-3-pyridinecarbonitriles [212] were synthesized.

Preclinical studies have assessed the best analogs among each series by assaying their IC50 values for the inhibition of PKC*θ* along with their metabolic stability in rat liver microsomes and their ability to block the production of interleukin-2 in stimulated human whole blood [213]. These compounds showed improved microsomal half-lives as well as decrease of interleukin-2 production. Molecules belonging to the category of thieno[2,3b]pyridine-5-carbonitriles are highly selective in nature. They are classified into two categories on the basis of substitution at their 2nd position, that is, 2- alkenyl, phenyl and 2-aryl derivatives [213]. A series of 2-alkenyl thieno[2,3b]pyridine-5-carbonitriles [214] and 4-(indol-5-ylamino)thieno[2,3-b]pyridine-5-carbonitriles were synthesized [215]. These compounds showed a decrease in interleukin-2 production by anti-CD3 and anti-CD28 activated T-cells derived from wild-type mice, with a reduced effect on activated T-cells from PKC*θ* knockout mice.

The experience with PKC*θ* inhibitors highlights several challenges for the future. PKC*θ* is an attractive therapeutic target, but clinically available inhibitors need to be more specific and selective against different PKC isoforms.

## 6. Conclusion

PKC*θ* is involved in many signaling pathways that control immune responses and other cellular activities, in normal physiology as well as certain disease states. Particularly, evidence highlights the T-cell activating role of PKC*θ* as an initiating event in many immunological disorders. Hence, the modulation of PKC activity becomes a challenge that, once overcome, will be useful in medical applications such as the regulation of autoimmune diseases and graft rejection. Accordingly, inhibitors of PKCs and PKC*θ* have been developed and tested in preclinical and clinical studies. Results are promising for the future development of more specific and selective inhibitors that can greatly enhance the treatment of several T-cell mediated diseases like asthma, arthritis, multiple sclerosis, autoimmunity, and organ transplantation.
